# Zeolites as nanoporous, gas-sensitive materials for in situ monitoring of DeNO_x_-SCR

**DOI:** 10.3762/bjnano.3.76

**Published:** 2012-09-26

**Authors:** Thomas Simons, Ulrich Simon

**Affiliations:** 1 RWTH Aachen University, Institute of Inorganic Chemistry, Landoltweg 1, D-52056 Aachen, Germany

**Keywords:** DeNO_x_-SCR, gas sensing, in situ, impedance spectroscopy, zeolite

## Abstract

In a proof-of-concept study we demonstrate in situ reaction monitoring of DeNO_x_-SCR on proton-conducting zeolites serving as catalyst and gas sensor at the same time. By means of temperature-dependent impedance spectroscopy we found that the thermally induced NH_3_ desorption in H-form and in Fe-loaded zeolite H-ZSM-5 follow the same process, while a remarkable difference under DeNO_x_-SCR reaction conditions was found. The Fe-loaded catalyst shows a significantly lower onset temperature, and time-dependent measurements suggest different SCR reaction mechanisms for the two catalysts tested. These results may help in the development of catalysts for the reduction of NO_x_ emissions and ammonia consumption, and provide insight into the elementary catalytic process promoting a full description of the NH_3_-SCR reaction system.

## Introduction

Zeolites are crystalline, nanoporous aluminosilicates composed of [TO_4_] tetrahedra (T = Si, Al). In H-form zeolites protons compensate the negative charge of the [AlO_4_] tetrahedra by forming bridging Si–OH–Al groups, so-called Brønstedt sites, leading to various technical applications in catalysis owing to the resulting acidic properties [1]. The acidity depends on the Si/Al ratio, called the silica modulus of a zeolite, and it can be measured by different measuring techniques, such as infrared (IR) spectroscopy [2], NMR spectroscopy [3], thermal analysis [4–6] and indicator method [7], as well as the amine titration method [8] and temperature-programmed desorption of ammonia (NH_3_-TPD) [9–11].

H-form zeolites are also applied in gas sensors [12–17]. They are proton conductors due to the mobility of the charge-compensating protons. By means of impedance spectroscopy (IS) [18–21] and quantum chemical calculations on H-ZSM-5 [22–23], we showed in previous works that protons can move through the zeolite framework by thermally activated hopping. The probability of proton hopping depends on the modulus and, thus, on the spatial distance between an occupied (protonated) and the spatially nearest unoccupied (deprotonated) Brønstedt site. In pure N_2_ as well as in N_2_ and O_2_ the presence of solvent molecules, such as H_2_O or NH_3_ in concentrations of about 10 ppm_v_ and above, leads to an increase of the proton mobility up to a temperature of 420 °C [24]. Above this temperature, desorption of the solvent molecules occurs. While the proton transport in the solvent-free zeolite can be described by means of classical hopping models, Grotthus-like and a vehicle-like transport has been identified in the presence of NH_3_, depending on the NH_3_ concentration and the temperature range, respectively. Hence, H-form zeolites can be applied as NH_3_-sensor materials [21,25–26].

The combination of gas sensitivity and catalytic activity renders zeolites as interesting materials for the study of the correlation of gas-sensing and catalytic properties in situ. This is of fundamental academic and technological interest, as it will potentially afford knowledge about the elementary reaction mechanisms [27]. For this purpose, the zeolite-mediated DeNO_x_-SCR (selective catalytic reduction of NO_x_ with NH_3_) may serve as a model reaction, and is of great relevance in the after treatment of diesel exhaust gas as well as in NO_x_-emitting technical plants. It relies on the conversion of NH_3_ with NO/NO_2_ and is catalysed by pure as well as by Fe- or Cu-loaded H-form zeolites [28–32]. Due to the NH_3_ sensitivity of the zeolites, the loading of the catalyst with NH_3_ as well as the depletion due to the SCR reaction may be directly monitored by IS. A first step in this direction was made by Kubinski and Visser, who performed AC-conductivity measurements at a fixed frequency of 4 Hz to monitor the NH_3_ loading of a base-metal zeolite catalyst (of proprietary composition) as a function of NH_3_ loading time, loading temperature and concentration [33]. Most recently, we were able to discriminate NH_3_ desorption from catalytic conversion under SCR-like conditions by IS on zeolite H-ZSM 5 with a Si/Al ratio of 80. We demonstrated that in situ monitoring of the NH_3_ conversion with NO_x_ becomes feasible when the zeolite is loaded with NH_3_ first and NO_x_ is applied to the gas phase afterwards [34].

In this study we compare for the first time H-ZSM 5 as the pure H-form with the Fe-loaded form, which is a state-of-the art catalyst in all NO_x_–NH_3_-SCR reactions with enhanced catalytic activity compared to the pure H-form. We assumed that direct observation of the SCR onset should be feasible and that the onset temperature for the Fe-loaded material would be significantly lower than for the H-form, while the ammonia desorption temperature for both materials should occur at the same temperature. We thus demonstrate that in situ monitoring of DeNO_x_-SCR by IS provides insight into the elementary catalytic process and, hence, may help in the development of a full description of the NH_3_-SCR reaction system.

## Results and Discussion

The zeolite materials were obtained from Süd-Chemie (München, Germany). Both materials show the same crystal morphology and have the same modulus *M* = 30, which was verified by AAS and EDX. Also the metal loading of the iron-containing material was quantified by EDX and XRF to be 4 wt % in agreement with the manufacturer’s data. According to the manufacturer the iron species are predominantly Fe_2_O_3_ nanoparticles (corresponding to the reddish colour of the material). However, the presence of individual ionic iron species has to be assumed, as well as monomeric iron.

**Figure 1:**
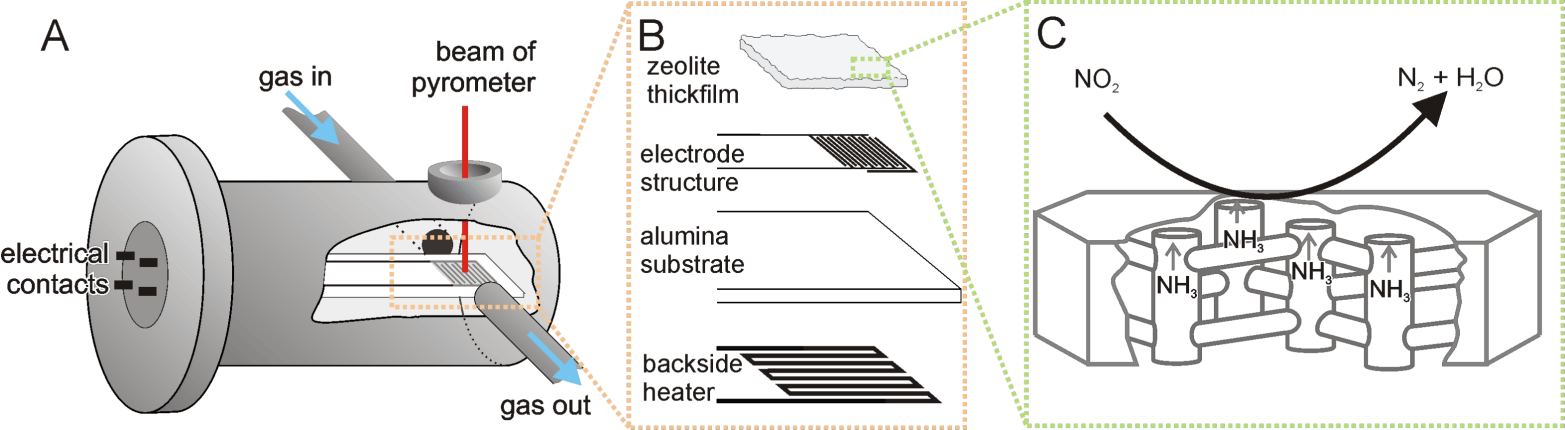
Experimental setup: (A) Schematic drawing of the measuring chamber; (B) Exploded drawing of the interdigital electrode structure; (C) Sketch of a zeolite crystal with simplified conversion scheme for the DeNO_x_-SCR reaction.

For in situ monitoring of the NH_3_ conversion with NO_x_, both zeolite materials were deposited as a thick film of 50 µm on interdigital electrodes (IDEs) on an actively heated sensor chip. The IDE structure is formed by screen-printed platinum electrodes on the front side of an alumina substrate with a width of 125 µm and a spacing of 150 µm. The whole electrode structure is about 8 mm in width and 9.5 mm in length. On the back side an integrated heating meander is applied (Figure 1; for electrode design see [24,35]).

For the measurements the IDE chip was placed in a stainless-steel measuring chamber (Figure 1), equipped with a ZnSe window for contactless temperature control by means of a spectral pyrometer (KT 19.82, Heitronics). For temperature regulation of the integrated heater a digital multimeter (Keithley 2400) was used. The gas flow through the chamber was controlled by mass-flow controllers (MKS 1179A and MKS 1259C operated by a four-channel controller MKS-647C; all MKS-Instruments). IS measurements were carried out with an impedance analyser combined with a dielectric interface (SI 1260 and SI 1296, both Solartron). To ensure that the samples were free of solvent molecules, the IDE chip was heated up to 500 °C in pure N_2_ and held at this temperature for 24 h before each of the measurement series was commenced.

To test our assumptions of different onset temperatures for both materials, we first performed a series of measurements to analyse the temperature-dependent impedance. Therefore, the nonsolvated samples were loaded with NH_3_ (100 ppm_v_ NH_3_ in N_2_) at room temperature for 1 h and afterwards heated up to 500 °C, in steps of 5 °C (100–300 °C), 10 °C (300–400 °C) and 20 °C (400–500 °C). To observe the thermal desorption of NH_3_ the complex impedance in a frequency range from 10^−1^ to 10^6^ Hz was measured under pure N_2_ at each temperature step, after an equilibration time of 15 min. In order to monitor the SCR reaction the heating procedure on the NH_3_ loaded zeolite was repeated in the presence of 10 ppm_v_ NO and 10% O_2_ with N_2_ as a balance, i.e., under SCR-like conditions. An unloaded sample in pure N_2_ was measured under the same heating conditions to serve as a reference.

**Figure 2:**
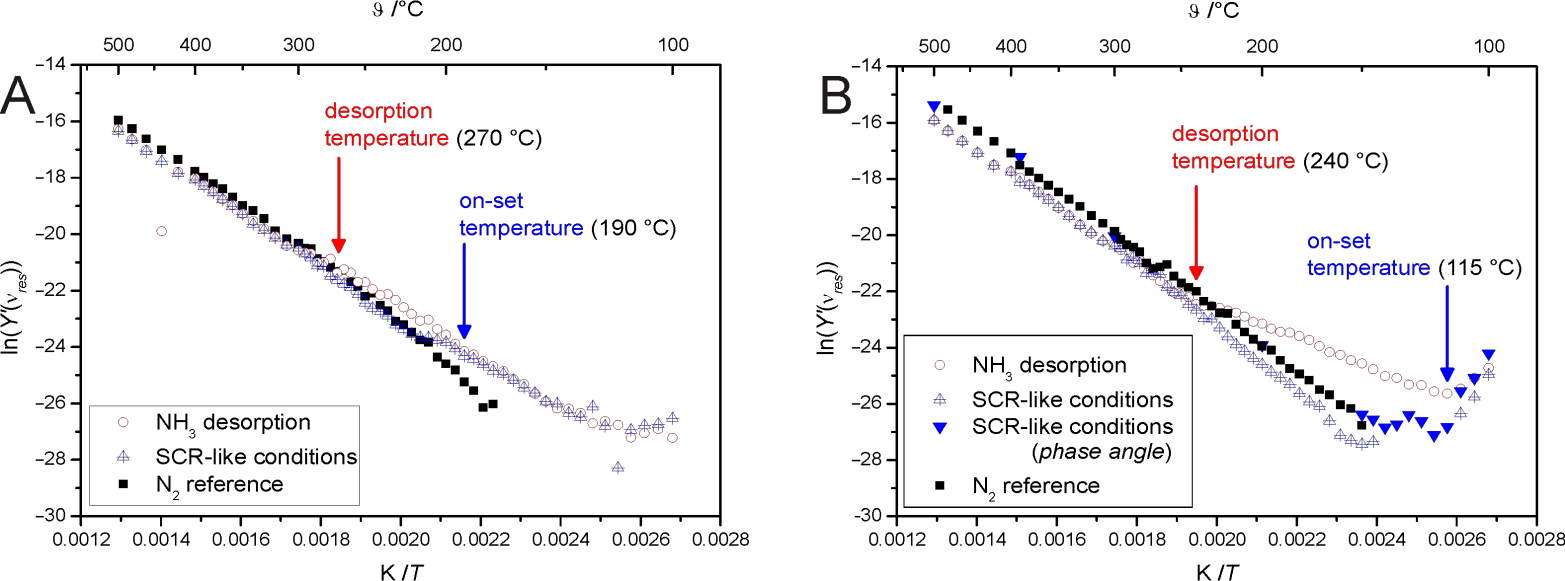
Arrhenius diagrams of pure proton zeolite ZSM-5 (A) and of Fe-loaded zeolite ZSM-5 (B).

As in our previous works on the ionic conductivity of zeolites [24,34], we evaluated the impedance data by analysing the spectral plot of the imaginary part of the modulus *M*″ (Equation 1). These plots typically show a distinct maximum with a resonance frequency ν*_res_*, which is shifted towards higher frequencies with increasing temperature. The corresponding real part of the complex admittance at the resonance frequency *Y*′(ν*_res_*) was subsequently determined and plotted in the form of an Arrhenius diagram (Figure 2).

[1]
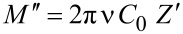


In this expression *C*_0_ is the geometric capacitance of the empty IDE. The relationship between the imaginary part of the modulus *M*″ and the conductance *G* is shown in Equation 2.

[2]
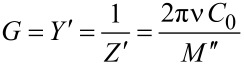


For the Fe-loaded sample, the conductivity was too low to gain clear modulus plots in the temperature range of 110–150 °C. Thus, we used the spectral plot of the phase angle φ (Equation 3) to determine the resonance frequency ν*_res_* in this temperature region.

[3]
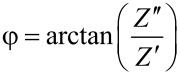


The relationship between the phase angle φ and the conductance *G* is shown in Equation 4.

[4]
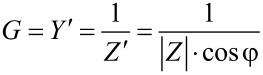


The resonance frequencies determined by using the phase angle φ are slightly different (up to a factor of two) from those determined from the imaginary part of the modulus *M*″. This is caused by a relaxation time distribution, which is not an ideal Debye relaxation in this system. This leads to an overrepresentation of the higher frequencies in the modulus plot and thus to a shift of ν*_res_* to higher frequencies [36].

The Arrhenius diagram of the H-form (Figure 2A) corresponds to the data of our earlier works [24,34]. While the unloaded sample (reference measurement under N_2_) shows a straight line, as is repeatedly observed for thermally activated proton hopping, the NH_3_-loaded sample shows increased conductivity below 270 °C due to the NH_3_-supported H^+^ motion. This solvent effect was described in detail in our previous works [24,34] for pure proton zeolites. Under SCR-like conditions at low temperatures the data are in agreement with the measurement data in NH_3_. With increasing temperature a decay of the conductivity appears at about 190 °C, which is significantly below the NH_3_ desorption temperature. This suggests NH_3_ conversion by NO_x_ with an onset temperature significantly below the desorption temperature of NH_3_. At temperatures of about 210 °C the conductivity again corresponds to that of the reference measurement.

The Arrhenius diagram of the Fe-loaded zeolite (Figure 2B) corresponds to the H-form for the reference measurement and also for the NH_3_ desorption measurement, with a comparable desorption temperature of about 240 °C. However, the data under SCR-like conditions differ from NH_3_ desorption already at very low temperatures. Only the very first data points of the SCR coincide with the data of NH_3_ desorption. At about 115 °C the conductivity decreases significantly to values approaching the reference measurement in pure N_2_. This reflects, again, an NH_3_ conversion by SCR reaction with an onset temperature significantly below the NH_3_ desorption temperature, but now significantly below the SCR onset temperature observed for the pure H-form.

**Figure 3:**
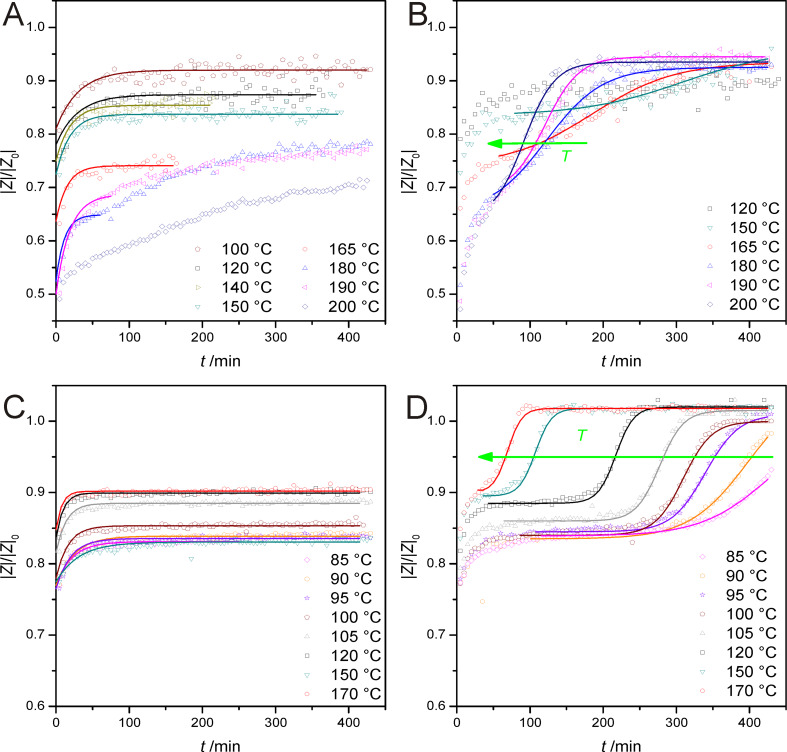
Time-dependence measurements: (A) NH_3_ desorption of pure proton form; (B) SCR-like conditions of the pure proton form; (C) NH_3_ desorption of the Fe-loaded form; (D) SCR-like conditions of the Fe-loaded form. The open symbols in the graph represent the measurement data, while the data fits are shown by the solid lines.

A second series of measurements were performed in order to measure the temporal evolution of the proton conductivity while the samples were kept at constant temperatures between 85 and 200 °C, after they have been loaded with NH_3_ for 45 min. After NH_3_ loading, the gas atmosphere was switched to N_2_, and the absolute value of the impedance *|Z|* at 1 Hz was recorded (in steps of 5 min) to monitor NH_3_ depletion in the zeolite layer. The same measurements were repeated under SCR-like conditions, and the data are presented in Figure 3 as time-dependence plots of the normalized absolute value of the impedance *|Z|/|Z|*_0_ at 1 Hz, whereas *|Z|*_0_ is the impedance at 1 Hz of a nonsolvated sample at the respective temperature.

The resulting curves for the NH_3_ desorption (Figure 3A for the pure proton form, Figure 3C for the Fe-loaded form) asymptotically increase but do not reach the impedance value for the nonsolvated state, because the applied temperatures were below the desorption temperature of the strongly bound NH_3_, of approx. 200 °C. At 180 °C and above the measurement curves in Figure 3A tend to adopt a sigmoidal shape. We attribute this to the temperature-driven desorption of strongly bound NH_3_, which does not take place at lower temperatures. Hence, in Figure 3A the asymptotical trend at 180 °C and above can only be observed at short reaction times. Therefore we restricted our data fitting to these parts of the curves. Based on this knowledge we limited all further measurements on the temperature range below 180 °C, in which only the more weakly bound NH_3_ species desorb, which are in the focus of the present study. In this temperature range both zeolite forms show similar curve shapes.

The resulting curves for the pure H-form under SCR-like conditions (see Figure 3B) also show an asymptotical increase for 120 and 150 °C. At higher temperatures the curves increase asymptotically for low reaction times, and with time a sigmoidal increase up to the impedance value for the nonsolvated state is observed. This distinct increase of the impedance values, in comparison to the NH_3_ desorption curve, points to a NH_3_ conversion by SCR reaction under these conditions. These characteristics were also observed in the curves for the Fe-loaded form under SCR-like conditions (see Figure 3D). For all temperatures between 85 and 170 °C the curve shows, at short reaction times, an asymptotical characteristic similar to the corresponding NH_3_ desorption curve. After a given reaction time for each temperature, the characteristic of the curve becomes sigmoidal and the impedance value increases up to the value for the nonsolvated state, but more pronounced than for the pure H-form sample. Although the Fe-loaded sample shows a slow onset of the SCR reaction at a temperature of 85 °C, observations at lower temperatures were not feasible due to the poor data quality (low signal-to-noise ratio, since the current-resolution limit of the impedance analyser is approached) at very low temperatures.

Hence, the Fe-loaded catalyst shows a NH_3_ loss by SCR reaction at significantly lower temperatures (start of the sigmoidal part of the curve is observed at 85 °C) than for the unloaded form, which is in accordance with the enhanced catalytic activity reported in literature. For both samples the sigmoidal part of the curves shows a shift of the inflection point to lower reaction times with increasing temperature, pointing to a thermal activation of the process.

To specify this thermal activation, we described all resulting curves for NH_3_ desorption and SCR reaction by mathematical curve fitting. For the asymptotic characteristic of the NH_3_ desorption, an exponential function (see Equation 5) was used.

[5]
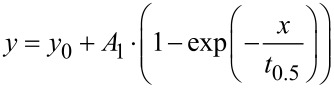


The value *t*_0.5_ describes the time that is needed to reach the half-maximum of the asymptotical increase. The sigmoidal part of the curves was described by using a Boltzmann-like function (Equation 6).

[6]
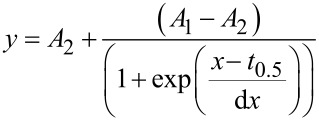


**Figure 4:**
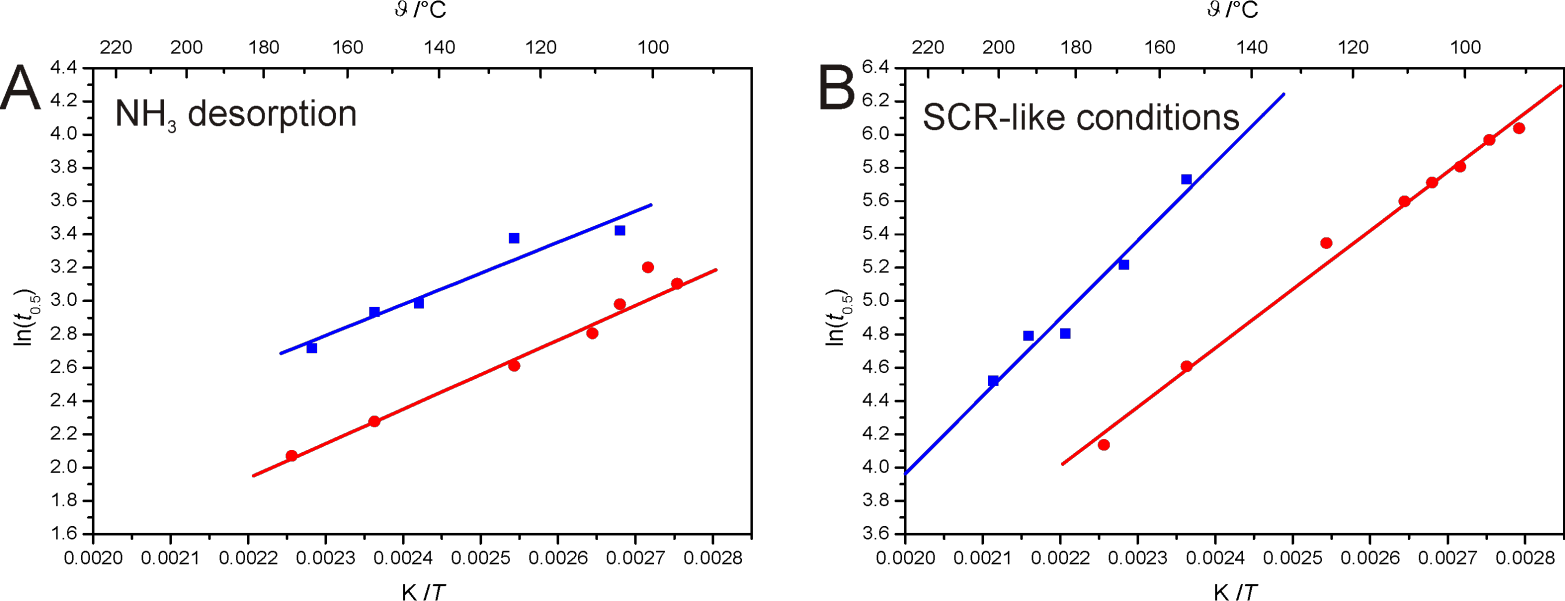
Comparison of the Arrhenius-like diagrams (blue squares: proton form; red dots: Fe-loaded form): (A) Comparison of the desorption processes; (B) Comparison under SCR-like conditions.

Here, the value *t*_0.5_ describes the time that is needed to reach the inflection point of the sigmoidal increase. The results of this mathematical curve fitting were plotted in an Arrhenius-like diagram by using the natural logarithm of *t*_0.5_ as the ordinate and the corresponding reciprocal temperature as the abscissa (Figure 4).

The results for the NH_3_ desorption are plotted in Figure 4A. The data points for the H-form and for the Fe-loaded form could be correlated by linear regression, respectively. An unpaired t-test was performed and the resulting P-value of about 0.196 indicates that the two slopes are not significantly different. This is in accordance with the assumption of the same process of NH_3_ desorption.

Figure 4B shows the values for the SCR reaction of both samples. Although the data points of both samples could be correlated by linear regression, the slopes of the regression lines clearly differ. An unpaired t-test led to P-values of less than 0.001 indicating a significant difference in thermal activation and thus a different reaction mechanism, which now deserves further and more quantitative investigation in our future work.

## Conclusion

In this work we were able to show that in situ reaction monitoring with proton-conducting zeolites serving as catalyst and sensor at the same time, becomes feasible by means of impedance spectroscopy. We found that the thermal NH_3_ desorption in a pure proton form and in a Fe-loaded zeolite H-ZSM-5 follows the same process, and that the thermal activation is not affected by the Fe-loading. Additionally we observed remarkable differences under DeNO_x_-SCR reaction conditions. The Fe-loaded catalyst shows a significantly lower onset temperature, and a comparison of time-dependence measurements at different temperatures suggests different SCR reaction mechanisms for the two catalysts tested. This is indicated by different thermal-activation characteristics, whereas no information about the underlying reaction has been derived so far.

While these results serve as a proof-of-concept, our follow-up experiments will concentrate on a more quantitative study involving spectroscopic analyses of the different molecular species involved. Another important aspect of our on-going work will be focused on different Fe species, their quantities, and their influence on the reaction. We strongly believe that different Fe species as well as different amounts of Fe will result in different reaction properties that can be detected by our impedance-based method. This would, however, require zeolite samples (catalysts) that contain only one type of Fe-species in a homogeneous manner. Thus, we expect to detect changes in the onset temperature and well as in the kinetic behaviour with such well-defined model catalysts.
